# Optimization of Emulsifier and Stabilizer Concentrations in a Model Peanut-Based Beverage System: A Mixture Design Approach

**DOI:** 10.3390/foods8040116

**Published:** 2019-04-04

**Authors:** Aggrey P. Gama, Yen-Con Hung, Koushik Adhikari

**Affiliations:** 1 Department of Food Science and Technology, University of Georgia, 1109 Experiment St, Griffin, GA 30223, USA; aggrey.gama25@uga.edu (A.P.G.); yhung@uga.edu (Y.-C.H.); 2 Department of Food Science and Technology, Lilongwe University of Agriculture and Natural Resources, P.O. Box 219, Lilongwe, Malawi

**Keywords:** visual stability index, centrifuge stability index, colloidal stability, rheology, mixture design, peanut beverage

## Abstract

Colloidal stability as well as physicochemical and rheological properties are among the critical determinants of the sensory quality of beverages. The present study investigated the effects of lecithin, xanthan gum, propylene glycol alginate, and their combinations on the colloidal stability and physicochemical/rheological properties of a model peanut-based beverage. A simplex centroid mixture design was applied, and the visual stability, centrifuge stability, physicochemical properties (soluble solids, pH, water activity, color), and rheological parameters (flow behavior and viscosity) of the samples were determined. All the evaluated parameters were significantly affected (*p* < 0.05) by the type and quantity of emulsifier or stabilizer used. At the 0.5% total usage level, the optimum stabilizer and emulsifier combination was that of 66% xanthan gum and 34% lecithin. A further increase of lecithin in the mixture caused a decrease in the colloidal stability of the sample. Irrespective of emulsifier and stabilizer type and quantity, all samples exhibited shear-thinning flow behavior, with samples containing xanthan gum being more pseudoplastic than the others. The prediction model for the visual stability index found in this study may be used by the industry to formulate similar beverages for better colloidal stability.

## 1. Introduction

Worldwide, the demand for health-promoting foods is growing due to an increase in the burden of non-communicable diseases and more consumer awareness in matters of diet and health [1]. Among such health-promoting foods are plant-based food products, especially from legumes like soy and peanuts. Over the past years, there has been more interest in legume-based beverages to replace or supplement cow milk consumption. For instance, several attempts have been made to develop peanut-based beverages [2,3,4,5,6,7,8]. As a result of such attempts, notable continual improvements in the physicochemical, nutritional, and sensory characteristics of the resultant peanut-based beverages have been achieved. However, there are still challenges, especially with sensory properties and colloidal stability of the peanut-based beverages. 

Depending on the ingredients, peanut-based beverages can be complex colloidal systems which in turn affect the sensory and rheological properties. In general, food products are mostly systems of mixed components (heterogeneous), and therefore often involve colloidal systems such as foams, emulsions, and suspensions, among others [9]. The stability of such colloidal systems is vital for the maintenance of desired quality and rheological characteristics of the food products [10,11,12]. A colloidal system is said to be stable if it shows little or no aggregation of particles and phase separation within a defined period. Unfortunately, food colloids are at a higher free energy level, and therefore they are thermodynamically unstable, with a tendency to aggregate and separate [13]. There are different modes of colloidal instability, and these include creaming and sedimentation due to the influence of gravity, aggregation due to attractive interparticle forces leading to either flocculation or coagulation, and coalescence if the interfacial film ruptures. A further mode of instability is Ostwald ripening or disproportionation due to differences in chemical potential of molecules in droplets or bubbles and those in the bulk phase [10,13].

Given the above, efforts have been made to prevent colloidal instability in food products through the use of emulsifiers and stabilizers. An emulsifier is an amphiphilic surface active chemical compound that facilitates the making of an emulsion and promotes short-term stability by rapidly adsorbing at the new interface created during emulsification [9]. Therefore, emulsifiers reduce interfacial tension and prevent immediate re-coalescence of the newly formed particles or droplets. On the other hand, a stabilizer confers long-term stability of the emulsion, once it is formed, through either adsorption or non-adsorption mechanism [10]. Little is known about the emulsifiers or stabilizers that would be effective in stabilizing peanut-based beverages, especially those using whole peanuts and not defatted peanut flour. Most emulsion stabilization systems in plant-based beverages involve the use of lecithin (emulsifier), propylene glycol alginate (both emulsifier and stabilizer), and xanthan gum (stabilizer) [14,15]. Therefore, the present study was undertaken to evaluate the effect of lecithin, propylene glycol alginate, xanthan gum, and their interactions on the colloidal stability and physicochemical/rheological properties of a model non-defatted peanut-based beverage. The emulsifier, stabilizer, or their combination that would be optimum in preventing colloidal instability of the product was also determined. The study could be beneficial to food product developers considering that determination of the optimum stabilizer, emulsifier, or stabilizer and emulsifier combination in a product provides economic benefits by decreasing the stabilizer and emulsifier concentrations in the formulation, as suggested by Dogan et al. [16]. Furthermore, the colloidal stability and physicochemical/rheological properties partly determine the sensory properties of food products, and therefore are determinants of consumer acceptability.

## 2. Materials and Methods

### 2.1. Preparation of the Model Peanut-Based Beverages

Peanut paste, dry non-fat milk powder, sugar, salt, propylene glycol alginate (PGA), xanthan gum (XG), and lecithin were used to prepare the model peanut-based beverages. Lecithin is an emulsifier, PGA is an adsorption stabilizer (surface-active), and XG is a non-adsorption stabilizer (non-surface active). Peanut paste was prepared by passing blanched medium roasted (lightness, L = 50 ± 1) Virginia peanuts (ICGV-SM 90704 variety, 45% oil content) through a colloidal mill (Model M-MS-3, Morehouse Industries, Los Angeles, CA, USA) two times. The peanuts were roasted at 162.8 °C (325 F) for 25 min using a convection oven (KitchenAid Superba, KEBS208), and then blanched using an Ashton peanut blancher (Model EX, Ashton Food Machinery Co., Newark, NJ, USA). The peanuts were obtained from ICRISAT Malawi, while the dry non-fat milk powder, sugar, and salt were obtained from Walmart Inc. (Bentonville, AR, USA). PGA and XG were obtained from TIC Gums (Belcamp, MD, USA) while lecithin was obtained from NOW® (Bloomingdale, IL, USA). 

The model peanut-based beverage contained 72.48% water, 15% roasted peanut paste, 7% sugar, 5% dry non-fat milk powder, 0.5% stabilizer, emulsifier, or their combination, and 0.02% salt. The concentrations of oil and protein in each sample were 6.75% and 5%, respectively. Eight samples, including a control, (S00, S01, S02, S03, S04, S05, S06, and S07) were prepared in duplicate. The respective ingredients were first mixed for 60 s in a blender and homogenized at 28,000 rpm for 60 s using an OMNI GLH-01 homogenizer (OMNI International, Kennesaw, GA, USA) before heating at 85 °C for 5 min on a hot plate. The heating was done under closed conditions while stirring with a magnetic stirrer to prevent evaporation loss and fouling. The beverages were cooled to 25 °C in a water bath, and all analyses were done with the samples at this temperature.

### 2.2. Determination of Stability Indices

Visual stability and centrifuge stability of the samples were evaluated. For visual stability, 100-mL samples were put in graduated cylinders for 24 h at 25 °C. The samples were then visually observed for separation, under gravitational force, into layers as done by Hinds et al. [5]. When a line of demarcation was observed, the ratio of the height of the top layer to a total height of beverage was determined and used to calculate visual stability index (VSI) through Equation (1). If no separation was observed, the visual stability index had a value of 1. A similar formula was used to calculate the centrifuge stability index (CSI), except that separation of 40-mL samples, in 50-mL corning tubes, was by centrifugal force of 3234× *g* (Centrifuge model 5804, Eppendorf AG, Hamburg, Germany). Readings were taken at seven different centrifugation durations (0, 1, 2, 3, 4, 5, and 6 min).

(1) $$\mathrm{VSI}\;\mathrm{or}\;\mathrm{CSI}\;=1\;-\;\left(\frac{\mathrm{Height}\;\mathrm{of}\;\mathrm{supernatant}}{\mathrm{Total}\;\mathrm{height}\;\mathrm{of}\;\mathrm{colloidal}\;\mathrm{mixture}}\right)$$

### 2.3. Measurement of Viscosity and Flow Behavior

Viscosity of 200-mL samples, at 25 °C, was measured using a programmable Brookfield digital viscometer (Model LV DV-II, Version 5.0, Brookfield Engineering Laboratories, Inc., Stoughton, MA, USA) fitted with an LV spindle No. 2. A 250-mL low form Pyrex beaker was used as a sample holder, as recommended by the LV DV-II viscometer operation manual. Viscosity readings were taken at six different spindle speeds (10, 20, 30, 40, 50, and 60 rpm). Five readings, at each spindle speed, were taken at three min of running the samples on the viscometer. Flow behavior was deduced graphically and by calculating the viscosity ratio as recommended in the Brookfield labs Inc. guide [17]. The viscosity ratio was the viscosity reading at 10 rpm to that of the 60-rpm spindle speed. Readings at 60 rpm were reported as viscosities of the samples because, under the test conditions, the 60 rpm was equivalent to a shear rate of 50 s^−1^ [17]. The value 50 s^−1^ is considered as the shear rate in the mouth [18,19].

### 2.4. Measurement of Color, pH, Water Activity, and Soluble Solids

The color of the samples was measured using a calibrated benchtop ColorFlex Spectrophotometer (HunterLab, Reston, VA, USA) with a D65 light source at a 45° viewing angle. Measurements for *L* (lightness), *a* (redness), and *b* (yellowness) were taken. The total soluble solids (°Brix), pH, and water activity (*a*_w_) of the samples were determined using a digital refractometer (ATAGO, PR-101, Atago USA, Inc., Bellevue, WA, USA), a pH meter (Accumet AE 150, Accumet Engineering, Inc., Westford, MA, USA), and a water activity meter (AQUALAB, Series 3, v2.3, METER, Pullman, WA, USA), respectively. All the measurements were done in triplicate.

### 2.5. Experimental Design and Statistical Analysis

A simplex centroid mixture design was used in this study. The design was generated using JMP package software (Version 5.0.1a, SAS Institute, Inc., Cary, NC, USA) and is given in Table 1. Equation (2) was fitted to predict VSI using the data obtained from the experiment. *β*_1_, *β*_2_, *β*_3_, *β*_12_, and *β*_13_ are the coefficients for each term, and X_1_, X_2_, and X_3_ are proportions of lecithin, propylene glycol alginate, and xanthan gum, respectively. Three-way interaction (X_1_X_2_X_3_) and two-way interaction (X_2_X_3_) terms were not included in the model because their respective effects were not significant (*p* > 0.05).

(2) $$Y=\beta_{1}X_{1}+\beta_{2}X_{2}+\beta_{3}X_{3}+\beta_{12}X_{1}X_{2}+\beta_{13}X_{1}X_{3}$$

The prediction equations were obtained using JMP Pro (Version 13.0, SAS Institute, Inc., Cary, NC, USA) for each response. Response surface plots were generated using Design-Expert software (Version 11.0, Stat-Ease Inc., Minneapolis, MN, USA). XLSTAT 2017 (ver. 19.01; Addinsoft, New York, NY, USA) was used to run a one-way analysis of variance (ANOVA) followed by Tukey’s honest significant difference test (HSD) to determine significant differences among the samples. Pearson’s correlation test was used to identify properties that had a significant association with VSI. 

## 3. Results and Discussion

### 3.1. Effect on Physicochemical Properties

The physicochemical properties of the samples are given in Table 2. The °Brix values of the samples varied between 13.7 and 15.8 °Brix. There were no statistically significant differences in the °Brix values of samples S02 and S03 compared to the control (S00). However, the °Brix values of samples S01, S04, S05, S06, and S07 were significantly higher than the control (*p* < 0.05). The pH values of the samples ranged from 6.11 to 6.26, and all samples had significantly lower pH values compared to the control. All samples had similar *a*_w_ readings (0.99) to that of the control, except sample S03, which had a significantly lower *a*_w_ (*p* < 0.05). However, all the samples had *a*_w_ readings above 0.95. Therefore, all the samples could be classified as having high water activity. Unlike the control and S02, which had similar lightness (*L*) values, the rest of the samples had significantly higher *L* values, with sample S06 being the lightest. Significant differences (*p* < 0.05) were also found in the yellowness (*b*) intensities of the samples with sample S06 having the highest yellowness intensity (18.52). However, all the samples had statistically similar intensities of redness (*a*) as the control. The observed effect of the emulsifiers and different stabilizers on the physicochemical properties was not peculiar. Other studies have also reported similar changes in °Brix, pH, *a*_w_, and color of beverages and other aqueous systems [16,20,21,22]. The molecular structure of the stabilizers or emulsifier, their water absorption capacity, and their interactions with each other and with the other ingredients in the beverage may have resulted in the observed effects on the physicochemical properties [16,21,23,24].

### 3.2. Effect on Flow Properties

**Figure 1:**
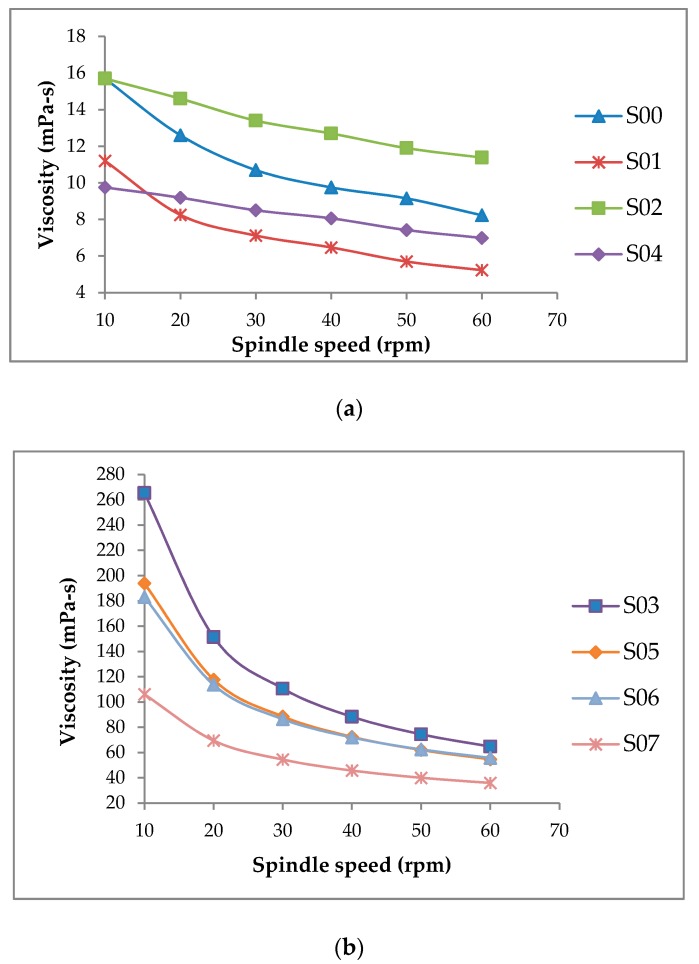
Change in viscosity as a function of shear rate (spindle speed). (**a**) Low-viscosity samples; (**b**) High-viscosity samples.

All the evaluated samples exhibited shear thinning flow behavior (Figure 1). However, deducing from the viscosity ratios (Table 2), the degree of shear-thinning of the samples was significantly different (*p* < 0.05). The viscosity ratios ranged from 1.38 to 4.10. All samples containing XG (S03, S05, S06, and S07) had relatively higher viscosity ratios than the other samples. The high viscosity ratios signified a high degree of shear-thinning. Other studies investigating the effect of gums on the rheology of beverages have also found shear-thinning flow behavior when XG is used [16,18,25]. High molecular weight and unique rigid rod-like conformation might contribute to the higher shear-thinning behavior of beverages thickened with XG-based thickeners [26,27]. In the present study, the degree of shear-thinning (pseudoplasticity) of the samples containing XG decreased with the addition of PGA. Other studies have found that unlike XG, PGA displays a low degree of shear-thinning in solution and promotes creaminess (sensation typical of fat-containing foods) without significant rheological changes [28]. Yilmazer et al. [29] reported that when XG was partially replaced by PGA, emulsions were more fluid-like and exhibited reduced viscosity. The relatively lower viscosities and viscosity ratios of samples containing PGA, in the present study, are therefore consistent with the findings of the other studies [28,29]. For the general population, a high degree of the shear-thinning flow behavior of the samples containing XG is a desirable property because it makes swallowing easier [30]. However, for consumers who might have disorders like dysphagia (a condition where swallowing is difficult), thicker but less shear-thinning products are desirable to avoid choking [25].

### 3.3. Effect on Viscosity and Colloidal Stability

The viscosity and VSI of the samples ranged from 5.23 to 65.70 mPa-s and 0.82 to 1.00, respectively (Table 2). All sample containing XG (S03, S05, S06, and S07) had relatively higher viscosity and were the most stable (VSI = 1). Therefore, it is not surprising that viscosity had a significant positive association with VSI (r = 0.74, *p* = 0.036). The significant positive association of VSI with viscosity confirms the mechanism through which non-adsorbing biopolymers (polysaccharides) stabilize food systems. The rates of creaming or sedimentation, Brownian motion, and particle collisions, respectively, are inversely related to the viscosity of the bulk phase [10,31]. However, the increase in viscosity of the bulk phase, by adding more thickener, does not always have a stabilizing effect. At some specific high biopolymer concentrations, depletion flocculation can be induced. It has been reported that when the separation distance between surfaces of colloidal particles, dispersed in a polymer solution, closely approaches the ‘diameter’ of the polymer molecules, the polymer chains are excluded from the gap between the particle surfaces [31]. The exclusion happens because the number of possible configurations that the polymer molecule, within the gap, can take is reduced resulting in a reduction in entropy. The reduction in entropy is against the second law of thermodynamics, and therefore a system will always strive to maximize its entropy [13]. After the polymer molecules have been depleted from the gap, there is relatively a higher polymer concentration in the bulk solution, causing an osmotic pressure imbalance. The solvent then diffuses from the gap into the bulk solution, thus drawing the particles much more closely to each other, which is technically called depletion flocculation [10,13]. Therefore, any attempts to keep on increasing the total gum concentration in the peanut-based beverage samples should be done with caution.

**Figure 2:**
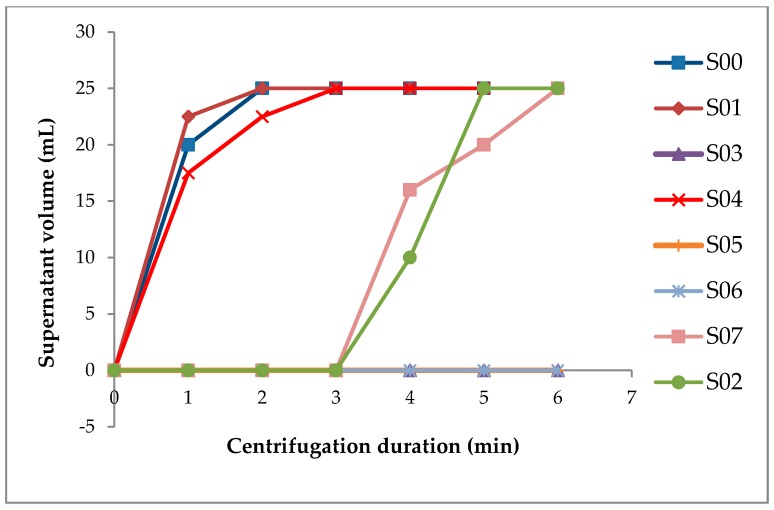
Separation behavior of the peanut-based beverages under centrifugal force of 3234× *g*.

Considering the nature of the evaluated samples, spoilage made it practically impossible to observe the samples, under room temperature conditions, for more than a day. Therefore, to further accelerate the separation process, a centrifugal force was applied, and a similar separation trend like that observed under gravitational force was also found. Samples containing XG (S03, S05, and S06) remained stable even after six min of centrifugation (Figure 2).

Sedimentation was the mode of colloidal instability in this study. All previous attempts to develop peanut-based beverages used defatted peanuts, probably to avoid creaming and coalescence among other reasons [2,3,4,5,6,7,8]. In the present study, non-defatted peanuts were used, but creaming was not observed in the samples including the control. The absence of creaming, even in the control sample, suggests that the homogenization efficiency was good and that there are other inherent emulsifiers in the beverage system. Considering the ingredients that were used, the likely emulsifiers may have been proteins like β-casein in milk. β-casein is a high molecular weight emulsifier but also a good stabilizer through its electrostatic and steric stabilization mechanisms [32,33]. The charge of β-casein gives the electrostatic repulsion while the protruding chains of the adsorbed β-casein provide the steric stability of emulsified fat particles [9,13].

### 3.4. Optimization and Prediction Model

Table 3 shows summary statistics of the prediction model for VSI. All model assumptions were satisfied. The model explained 99.5% (Adjusted R^2^ = 0.985) of the variation in VSI of the samples and was the best fit since the RSME (0.0043) was less than 0.05. Therefore, maintaining all the other factors constant, the model may be used to predict VSI, based on the different stabilizer and emulsifier combinations. 

**Figure 3:**
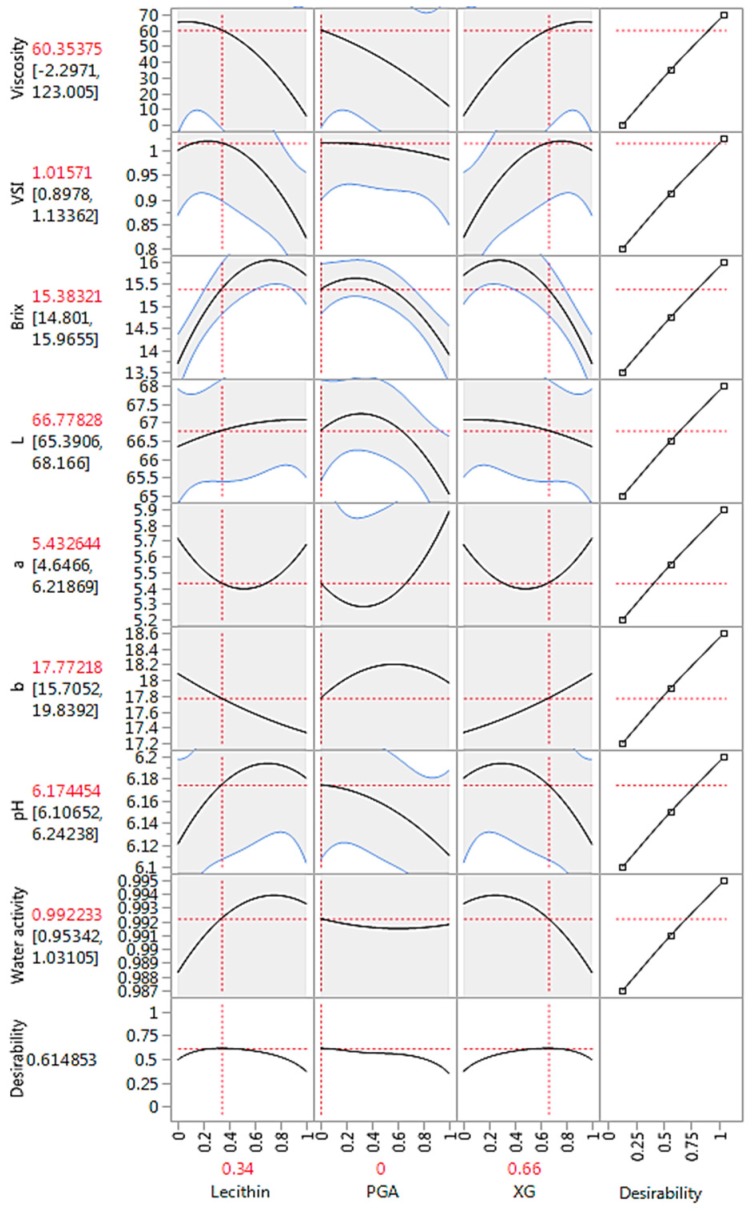
Optimal prediction profiles of the effect of the different emulsifier and stabilizers on the various properties of the model peanut-based beverage. VSI = visual stability index; *L* = lightness; *a* = redness; *b* = yellowness; PGA: propylene glycol alginate; XG: xanthan gum.

**Figure 4:**
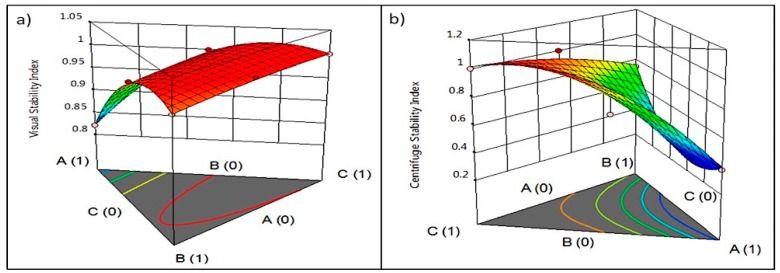
Ternary and surface contour plots showing the effect of the stabilizer-emulsifier systems on (**a**) visual stability index (VSI) and (**b**) centrifuge stability index (CSI) of the model peanut-based beverage. A = lecithin; B = PGA; C = XG.

As previously discussed, the parameter estimates (coefficients) values of the model terms also confirm that XG had a greater positive effect on VSI, likely as a result of the significant increase in viscosity of the samples. Although viscosity had a significant positive correlation with VSI, a viscosity of above 65 mPa-s would make the product too thick, and therefore not ideal for a beverage. Therefore, considering both VSI and viscosity, a combination of lecithin (0.34) and xanthan gum (0.66) was optimal (Figure 3). The prediction model for VSI is also consistent with this finding (Table 3). Although lecithin on its own had a negative effect on VSI (Figure 4a) and CSI (Figure 4b), it moderated the effect of XG, and their combination had a positive effect on the colloidal stability of the samples.

## 4. Conclusions

The colloidal stability and physicochemical/rheological properties of the peanut-based beverages were affected by the amount and type of the stabilizer or emulsifier used. In this study, a mixture design was applied to obtain the optimum stabilizer and emulsifier combination for the peanut-based beverage. The optimum stabilizer and emulsifier combination was that of 66% xanthan gum and 34% lecithin at the 0.5% usage level by weight of the beverage. The use of the lecithin alone resulted in a decrease in viscosity, VSI, and CSI of the samples. The prediction model found in this study may be used in industry to achieve colloidal stability of similar beverages. However, further studies should be done to determine the sensory characteristics and consumer acceptability of the stabilized peanut-based beverages. 

## Figures and Tables

**Table 1:** Simplex centroid mixture design for the emulsifier and stabilizers.

Sample Codes	Gum and Emulsifier Proportions (%)		
	Lecithin (*X*_1_)	PGA (*X*_2_)	XG (*X*_3_)
S00 (Control)	0	0	0
S01	100	0	0
S02	0	100	0
S03	0	0	100
S04	50	50	0
S05	50	0	50
S06	0	50	50
S07	33.3	33.4	33.3

PGA: propylene glycol alginate; XG: xanthan gum.

**Table 2:** Physicochemical and rheological properties of the model peanut-based beverages.

Sample	pH	°Brix	*a*_w_	Viscosity (mPa-s)	VSI	Viscosity Ratio	Color		
							*L*	*a*	*b*
S00	6.26 ± 0.01 ^a^	14.00 ± 0.14 ^c^	0.99 ± 0.00 ^a^	8.23 ± 0.04 ^f^	0.92 ± 0.00 ^c^	1.91 ± 0.01 ^f^	65.12 ± 0.08 ^d^	5.66 ± 0.25 ^ab^	17.51 ± 0.08 ^de^
S01	6.18 ± 0.01 ^b^	15.65 ± 0.07 ^a^	0.99 ± 0.00 ^a^	5.23 ± 0.04 ^h^	0.82 ± 0.00 ^d^	2.14 ± 0.01 ^e^	67.06 ± 0.08 ^b^	5.67 ± 0.02 ^ab^	17.32 ± 0.06 ^e^
S02	6.11 ± 0.01 ^d^	13.90 ± 0.14 ^c^	0.99 ± 0.00 ^a^	11.38 ± 0.03 ^e^	0.98 ± 0.00 ^b^	1.38 ± 0.00 ^g^	65.04 ± 0.12 ^d^	5.88 ± 0.05 ^a^	17.95 ± 0.03 ^bc^
S03	6.12 ± 0.01 ^d^	13.70 ± 0.14 ^c^	0.98 ± 0.00 ^b^	65.70 ± 0.01 ^a^	1.00 ± 0.00 ^a^	4.10 ± 0.00 ^a^	66.33 ± 0.08 ^c^	5.71 ± 0.17 ^ab^	18.07 ± 0.10 ^b^
S04	6.13 ± 0.01 ^cd^	14.80 ± 0.14 ^b^	0.99 ± 0.00 ^a^	6.99 ± 0.01 ^g^	0.98 ± 0.00 ^b^	1.39 ± 0.00 ^g^	66.18 ± 0.16 ^c^	5.59 ± 0.04 ^ab^	17.80 ± 0.02 ^bcd^
S05	6.19 ± 0.01 ^b^	15.80 ± 0.28 ^a^	0.99 ± 0.00 ^a^	54.55 ± 0.07 ^c^	1.00 ± 0.00 ^a^	3.55 ± 0.00 ^b^	66.96 ± 0.03 ^b^	5.42 ± 0.00 ^ab^	17.71 ± 0.03 ^cd^
S06	6.16 ± 0.01 ^bc^	15.40 ± 0.00 ^ab^	0.99 ± 0.00 ^a^	55.68 ± 0.04 ^b^	1.00 ± 0.00 ^a^	3.29 ± 0.00 ^c^	67.53 ± 0.06 ^a^	5.32 ± 0.22 ^b^	18.52 ± 0.13 ^a^
S07	6.16 ± 0.01 ^bc^	15.70 ± 0.14 ^a^	0.99 ± 0.00 ^a^	35.89 ± 0.01 ^d^	1.00 ± 0.00 ^a^	2.95 ± 0.00 ^d^	66.98 ± 0.01 ^b^	5.25 ± 0.08 ^b^	17.85 ± 0.08 ^bc^

^abcdefgh^ Common letter in each column indicates no statistical difference at 5% significance level. *a*_w_ = water activity, VSI = visual stability index; *L* = lightness; *a* = redness; *b* = yellowness.

**Table 3:** Effect of emulsifier and stabilizers on the visual stability index (VSI) of the peanut-based beverages.

Term	Estimate	95% CI	χ^2^	*P*	VIF
X_1_ (Lecithin)	0.82	0.81–0.84	6086.72	<0.0001	0.9913
X_2_ (PGA)	0.98	0.97–1.00	8678.29	<0.0001	0.8295
X_3_ (XG)	1.00	0.99–1.02	9017.56	<0.0001	0.8295
X_1_X_2_	0.30	0.23–0.37	38.53	<0.0001	20.9249
X_1_X_3_	0.34	0.27–0.41	48.86	<0.0001	20.9249
**Model Fit Statistics**					
R^2^	0.9950				
Adjusted R^2^	0.9849				
RMSE	0.0043				

CI = confidence interval; *P* = Probability of Type I error; VIF = variance inflation factor; RMSE = square root of the variance of the residuals.
